# Covid-19: A Dynamic Analysis of Fatality Risk in Italy

**DOI:** 10.3389/fmed.2020.00185

**Published:** 2020-04-30

**Authors:** Marco Iosa, Stefano Paolucci, Giovanni Morone

**Affiliations:** Clinical Laboratory of Experimental Neurorehabilitation, IRCCS Fondazione Santa Lucia, Rome, Italy

**Keywords:** SARS-CoV-2, Corona virus, case fatality ratio, epidemiology, rehabilitation

## Abstract

Italy was the second country in the world to face a wide epidemic of Covid-19 after China. The ratio of the number of fatalities to the number of cases (case fatality ratio, CFR) recorded in Italy was surprisingly high and increased in the month of March. The older mean age of population, the changes in testing policy, and the methodological computation of CFR were previously reported as possible explanations for the incremental trend of CFR, a parameter theoretically expected to be constant. In this brief report, the official data provided by the Italian Ministry of Health were analyzed using fitting models and the linear fit method approach. This last methodology allowed us to reach two findings. The trend of the number of deaths followed a 1–3-day delay of positive cases. This delay was not compatible with a biological course of Covid-19 but was compatible with a health management explanation. The second finding is that the Italian number of deaths did not increase linearly with the number of positive cases, but their relationship could be modeled by a second-order polynomial function. The high number of positive cases might have a direct and an indirect effect on the number of deaths, the latter being related to the overwhelmed bed capacity of intensive care units.

## Introduction

The severe acute respiratory syndrome coronavirus 2 (SARS-CoV-2) has developed worldwide into a pandemic (1, 2). There is a wide clinical debate on the different strategies required to minimize deaths and a political one on the economic impact of those strategies, but minimizing both fatalities and cost is proving to be quite difficult (3).

In China, the epidemic seemed to be effectively contained by quarantine, social distancing, and the isolation of the infected population. Conversely, on March 2020, the spread of Corona Virus Disease (Covid-19) in Italy largely increased despite the restrictions put in place by the Government. In Italy, the first case of SARS-CoV-2 was diagnosed in Lombardy region on the 20^th^ of February 2020 (4). Only 1 month later, the number of deaths due to the Covid-19 recorded in Italy was the highest globally, even higher than that documented in China, and this was only recently exceeded by United States.

Many different mathematical models have been proposed to help governments to decide on what health policies they should follow. Some models have been based on an exponential curve for fitting the number of infected cases and deaths. Although mass media reported this initial exponential trend, it was conceivable to expect a deviation from that—rather than a plateau—followed by a progressive decrement, according to a bell-shaped curve (5). A recent study based on data recorded up to the 8th of March hypothesized for Italy a trend similar to that observed in the Hubei Province in China, and it predicted a peak of cases at around the 10th of April (5).

Anderson et al. (3) have developed an illustrative simulation of the transmission model of Covid-19, showing that social distancing could flatten the curve of positive case frequency, retarding and reducing the peak of the curve estimation in case of no social restrictions (3). This theoretical modeling, reported by many mass media, suggested that a delay in contagions may reduce the number of deaths. This hypothesis was based on the idea that the number of beds in intensive care units (ICUs) could be sufficient only for a flattened curve of positive case frequency. Otherwise, if the number of severely affected patients exceeded that of beds in ICUs, the number of deaths could dramatically increase.

At the beginning, the Italian case fatality rate (CFR) seemed to be similar to that of China, initially fixed at 2.3% (5). The case fatality rate is the ratio of deaths caused by a given disease calculated on the total number of cases that the disease generated in a specific time period (6). Updated with the new data from the 29th of March, the Italian CFR exceeded the 10%. In a comparison report, a possible explanation was provided by the higher mean age of the Italian population compared with the Chinese one (7). But this may be only a partial explain of the difference in the case fatality rate of Covid-19 in Italy with respect to China. An older population, such as the Italian one, may suffer from comorbidities, which increase the risk of death and hence the CFR (7). However, the Italian CFR has been higher than the Chinese one even after being corrected for age: in patients older than 80 years, CFR was 20.8% in Italy, and 14.8% in China (7). Furthermore, the mean older age of people did not explain the incremental trend for CFR within the Italian population during the month of March.

The authors of that research suggested also other possible explanations for the high CFR, mainly related to the methodological differences in case recording and case testing (7). In the early phase of the epidemic, Italy carried out an extensive testing strategy by collecting swabs of both symptomatic and asymptomatic contacts of the infected patients, as was done in China. Then, the Italian Ministry of Health issued more stringent testing policies, prioritizing tests for patients with severe clinical symptoms who required hospitalization. This could have caused an increase in the computed value of CFR for the underestimation of the number of the asymptomatic or mildly affected patients for whom the tests were often not administered. It means that, in Italy more than in other countries, the full denominator of CFR remains unknown because asymptomatic cases or patients with mild symptoms might not be tested and hence will not be identified.

Recent studies have faced the problem of a correct computation and interpretation of CFR related to Covid-19. One of them suggested, in this dynamic situation, to estimate the CFR as the number of deaths on the number of infected patients evaluated 2 weeks before (8). This delay was suggested to be helpful for taking into account the incubation period and the median time from onset of symptoms to death (9, 10).

A recent report, using a delay-adjusted CFR of 1.38% (computed from a previous large study conducted in China), estimated that less than the 5% of the contagions in Italy were actually diagnosed^1^. However, it is noteworthy that the Italian policy change on tests occurred on the 25th of February when the Italian CFR was 3.4% and had then continued to increase, hitting 10% only 1 month later on the 25th of March.

Italy was the first Western country with a wide spread of Covid-19, and it could be important, for other countries, to analyze in depth the Italian case. The Italian CFR increased day by day, despite, from a theoretical point of view, the CFR being expected to be constant (6). A constant CFR means that the number of deaths proportionally (linearly) increase with the number of cases. The above studies seemed to suggest that CFR was only miscomputed because the more severe cases the clinicians need to bring assistance to the less time they have to test non-severe cases, causing an apparent increase of CFR^1^ (8).

**Figure 1:**
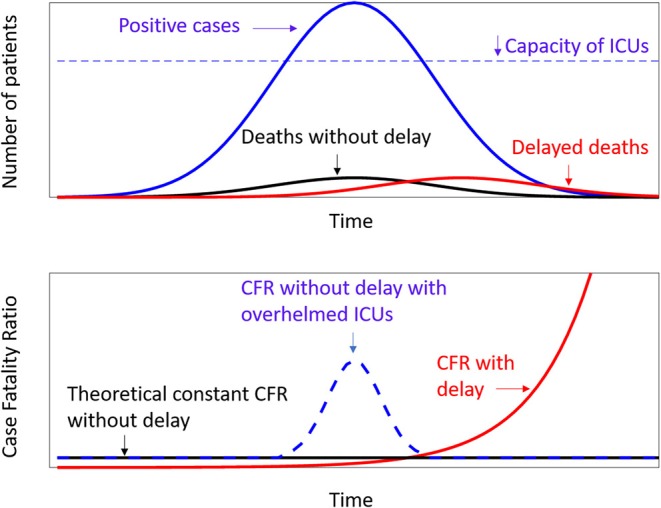
Theoretical models. Above are the frequency curves of positive cases (blue line), deaths (black line), and deaths with a delay with respect to the positive cases (red line). The dotted line represent the capacity of Intensive Care Units, as hypothesized by Anderson et al. (3). Below the relevant values of Case Fality Ratio according to the above distributions.

In the present study, mathematical models were used to test if the high Italian CFR was only apparent because it was related to an underestimation of positive cases or if it represents a real increment of Covid-19 lethality, maybe related to the difficulties of the Health National System to manage many cases in a short period and in a small region as occurred in the north of Italy. These possibilities have led to the different theoretical scenarios depicted in Figure 1. The CFR computed day by day could be high due to the need to take into account a biological delay of about 14 days between deaths and the recorded number of positive cases (8) or for the insufficient number of beds into ICUs. In the former case, there is a statistical problem, whereas, in the latter case, the health policy of other countries should take into account the Italian lesson for Covid-19. The aim of this study was to provide a deeper insight into the Italian CFR, testing the hypothesis that the number of deaths increased more than linearly with the number of positive cases.

## Materials and Methods

In this study, the data officially provided by the Italian Ministry of Health and Istituto Superiore di Sanità ^2^ were used to monitor the increment of cases of contagion and death related to Covid-19 in Italy. Data were collected from the 24th of February to the 29th of March 2020 (Supplementary Table 1). Polynomial, logistic, and bell-shaped functions were applied to fit the data. The equation of a bell-shaped function was the following

$$\begin{array}{l} f=a•e^{-\frac{{\left(x-m\right)}^{2}}{s^{2}}} \end{array}$$

The adjusted coefficient of determination (R^2^) was preferred to the raw one to assess the goodness of the fitting models independently by the number of their coefficients.

The approach of the Linear Fit Method (LFM) was used to compare the number of cases and that of deaths. This method was previously validated for assessing the waveform similarity in clinical data. The LFM relies on the idea of plotting one dataset vs. another one to compare the similarity of their waveforms, such as the contemporaneity of their peaks (11).

In the rapid evolution of the pandemic of Covid-19, the day-by-day CFR was computed. It means that, for each day, the CFR was the percentage of deaths on the number of actually positive patients plus dead patients plus discharged patients.

The theoretical scenarios are depicted in Figure 1, which reports the case of a constant CFR as theoretically expected (6) and that of a CFR computed to take into account a biological delay (8). A third case is reported, related to a dynamic perspective of CFR taking into account a potential increase in the period in which the number of severe cases overwhelmed the capacity of Intensive Care Units (ICUs), which was the worst-case scenario hypothesized byAnderson et al. (3).

## Results

### Analysis of the Ongoing Epidemic of Covid-19 in Italy

**Figure 2:**
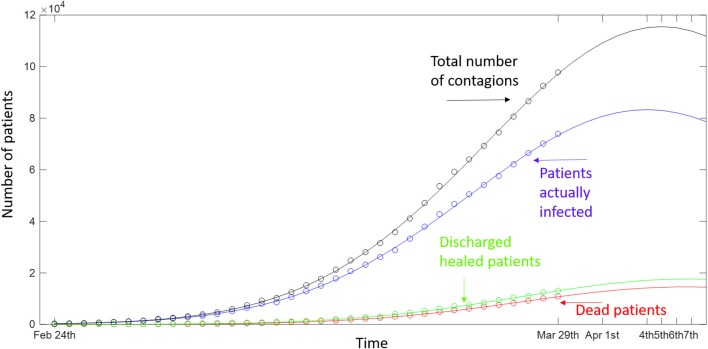
The day-by-day Italian data (dots) for positive cases (blue), dead patients (red), discharged patients (green), and the total sum of these cases (black). The continuous line represents the bell-shaped functions fitting the data.

The bell-shaped models of Figure 2 show that the number of positive cases in Italy is still increasing day by day, as is that of deaths. Although a prediction is very difficult, these models have exhibited very high values for the adjusted coefficient of determination R^2^ (0.999 for actually positive, total infected, and dead patients, whereas it was 0.998 for discharged patients). Independently by the goodness of the predictions, the trend of deaths seems to follow that of infections, with a delay of about 3 days.

### Analysis of the Italian CFR

**Figure 3:**
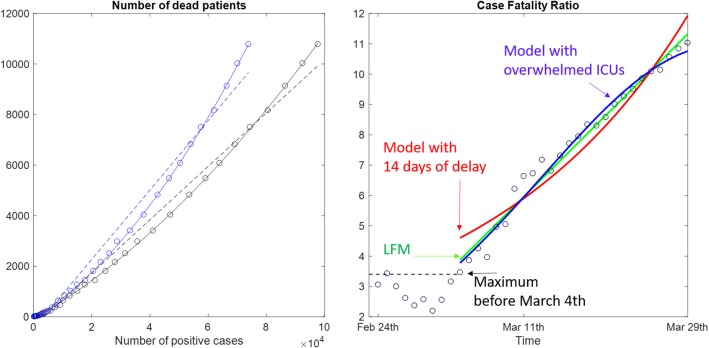
On the left, the number of deaths were plotted vs. the number of actually positive (blue dots) and the total number of contagions (black dots). Linear (dotted lines) and second-order polynomial (solid lines) fits were reported. On the right is the temporal trend of the case fatality ratio evaluated day by day (dots) and a linear interpolation (green line), a model with delayed death (red line), and a model with an bell-shaped curve superimposed to a constant CFR (blue line).

The linear fit method approach has allowed us to compare the trends of real data, as reported in Figure 3. The number of dead patients increased with the increment of infected patients (left panel). As clearly shown by the data, this increment has a second-order polynomial trend more than the expected linear one. When the CFR was computed (right panel of Figure 3), an initial quite constant low value of CFR was observed, and it was followed by a progressive increment. In fact, in the first 9 days of data collection, the Italian CFR was roughly constant and lower than 3.5%. It then started to increase. The linear increment computed using the LFM showed that *R*^2^ = 0.977. The model, based on a theoretical biological delay of 14 days in the computation of deaths, showed a lower value *R*^2^ = 0.916. Furthermore, this model had a concavity opposite to that revealed by data. Conversely, in this phase, a bell-shaped increment related to the overwhelmed ICUs showed that *R*^2^ = 0.980 in fitting the data. This last model coincided with a double bell-shape model with a delay of only 1 day between positive tests and deaths.

## Discussion

Mathematical models and parameters are often used in epidemiology to generate insight into the transmission dynamics of infectious diseases and to assess the potential impact of the different intervention strategies.

First of all, Italian data and our models supported the theoretical prediction that the Italian trend of infected patients could be similar to that one of China. This prediction was previously suggested by Remuzzi and Remuzzi on the basis of Italian data recorded up to the 8th of March upon which a tend similar to that observed in the Hubei Province, China, was applied (5). Our results indirectly suggested that the Italian interventions, mainly based on the social distancing, have been effective in reducing the speed of contagions, as occurred in China. These restrictions seemed to reduce the increment of infected patients (often incorrectly reported as an exponential growth), preventing the intensive care units in the rest of Italy from being overwhelmed as occurred in Lombardy (4).

However, the resulting Italian CFR was very high and progressively increased throughout March. This could be due to a miscomputation of CFR^1^ (8). However, Figure 3 clearly shows the number of deaths increased following a second-order polynomial function with respect to the number of positive cases. In a theoretical stationary situation, CFR is expected to be constant, meaning that the number of deaths proportionally (linearly) increased with the number of positive cases. But the high number of positive cases that occurred in Lombardy in a small period might have overwhelmed the ICUs, having a secondary effect on the number of deaths in that Italian Region.

In the case of Covid-19, the case fatality rate might be relevant for optimizing a health policy. Many recent studies investigating this CFR have tried to explain the high value recorded in Italy and progressively in other Western Countries^1^ (7, 8). Our study showed that the Italian data had a different and unexpected second-order increment of the number of deaths related to Covid-19 with respect to the relevant number of infected patients. Some authors have suggested that it could be due to the change in testing policy (7), but the increasing trend occurred even after this change. Other authors have suggested a correction in CFR computation for taking into account the time of incubation and worsening (8), but it seemed to fail in modeling the Italian data. In fact, our results, obtained with different data analysis, seemed to show a delay ranging from 1–3 days between the curve of positive cases and that of deaths. Furthermore, the concavity of the 2-week delayed CFR seemed to be opposite to that of data.

The small delay found in our analyses was not compatible with a biological explanation, but it could be compatible with a health management explanation. This hypothesis seemed to be confirmed by a bell-shaped increment of deaths related to the difficulties of ICUs in managing a high number of patients with severe symptoms.

It is possible that, although all the possible miscomputation of CFR could be related to an underestimation of positive cases, the Italian CFR was affected by what happened in Lombardy Region, the region most infected. It was a scenario of an unexpected high number of cases, most of them recorded in a small area and in a short period of time (about 5 weeks).

The Italian Health Policy was conceivably effective in attenuating the Lombardy trend in the other Regions, reducing the velocity of contagions thanks to the imposed social distancing. Furthermore, in Lombardy and in other regions, the number of beds in ICUs was increased. This possible explanation did not exclude that the high CFR was also due to an underestimation of positive cases. The emergency might also have leaded clinicians to focus on severe cases, progressively applying the reduction of tests in mildly affected and asymptomatic people (7). Both these explanations, related to health policy, could be concomitant with the progressively increased high value of Italian CFR.

Many other countries are now facing the emergence of Covid-19, and the computation of CFR could be misleading, even taking into account the biological delay. In an emergency and rapidly changing scenario such as the Italian one, the CFR should be interpreted from a dynamic perspective, as it is potentially affected by many changing variables with effects that are not necessarily linear. Direct and indirect effects of a wide contagion should be taken into account. The analysis of the evolution of the Italian CFR trend could be of help to further develop a suitable health policy in other countries. For example, in further studies, it could be important to assess the complementary value of CFR, which is related to recovered patients. There could be an important percentage of them needing rehabilitation of motor and respiratory functions. Some of these patients may not be able to wait for the end of emergency, but the health policy should face the problem of rehabilitation with a respect for safety. Even unaffected older people may have motor deficits related to the long period spent at home. Another aspect could be the psychological effects of Covid-19 in recovered patients, including the fear of being infected or the psychological effects of social distancing in uninfected people (12).

## Data Availability Statement

The datasets for this study can be found in the Repository of Frontiers and also in the official site of Italian Ministery of Health (www.salute.gov.it).

## Ethics Statement

Ethical review and approval was not required for the study on human participants in accordance with the local legislation and institutional requirements. Written informed consent for participation was not required for this study in accordance with the national legislation and the institutional requirements.

## Author Contributions

MI performed all the data analysis and wrote the first draft of the manuscript. GM and SP provided important clinical content to the manuscript and supervised the study.

## Conflict of Interest

The authors declare that the research was conducted in the absence of any commercial or financial relationships that could be construed as a potential conflict of interest.

## Footnotes

^1^https://cmmid.github.io/topics/covid19/severity/global_cfr_estimates.html

^2^http://salute.gov.it

## Supplementary Material

Supplementary Table 1 Official Italian data related to Covid-19 provided day-by-day by Italian Ministry of Health from the 24th of February to the 29th of March 2020.
